# Paradigm Change? Cardiac Output Better Associates with Cerebral Perfusion than Blood Pressure in Ischemic Stroke

**DOI:** 10.3389/fneur.2017.00706

**Published:** 2017-12-22

**Authors:** Hannah Fuhrer, Matthias Reinhard, Wolf-Dirk Niesen

**Affiliations:** ^1^Department of Neurology, University Medical Center, University of Freiburg, Freiburg, Germany; ^2^Department of Neurology, Medical Center Esslingen, Teaching Hospital of the University of Tuebingen, Esslingen, Germany

**Keywords:** cardiac index, cardiac output, cerebral perfusion, stroke, perfusion imaging

## Abstract

**Introduction:**

In patients with acute ischemic stroke, penumbral perfusion is maintained by collateral flow and so far is maintained by normal mean arterial pressure (MAP) levels. Since MAP is dependent on cardiac function, optimization of cardiac output might be a valuable hemodynamic goal in order to optimize cerebral perfusion (CP).

**Methods:**

Cerebral perfusion was assessed by transcranial color-coded duplex and transcranial perfusion sonography in 10 patients with acute large hemispheric stroke. Time-to-peak (TTP) values of defined regions of interest (ROI) within the middle cerebral artery (MCA) territory were assessed bilaterally in addition to mean flow velocities of the MCA. *Via* semi-invasive advanced hemodynamic monitoring systemic hemodynamic parameters were assessed, including MAP and cardiac index (CI). Patients received sonographic follow-up after optimizing CI.

**Results:**

TTP values of the deeply located ROIs of the non-affected as well as the affected hemisphere correlated highly significantly with CI (in affected side *r* = −0.827, *p* = 0.002; and in non-affected side *r* = −0.908, *p* < 0.0001). This demonstrates dependence of CP on CI, while correlation with MAP was not detected. Neither CI nor MAP revealed significant correlation with MCA velocity.

## Introduction

Restoring penumbral perfusion is the key target of therapeutic efforts in acute ischemic stroke. The penumbra is characterized by reduced perfusion with functional impairment but without immediate irreversible damage (1, 2). So far, in cases of insufficient or unsuccessful vessel recanalization, maintaining normal to higher mean arterial pressure (MAP) is the goal to preserve penumbral perfusion. MAP is expected to be related to cerebral perfusion (CP), as constant cerebral blood flow (CBF) is maintained over a wide range of MAP due to vessel autoregulation (3).

Furthermore, MAP is expected to correlate with cardiac output (CO), since MAP is the product of CO and systemic vascular resistance [SVR; (4)]. In order to increase MAP, CO according to the Frank-Starling curve and SVR are enhanced *via* fluid and/or catecholamine administration (5). Former studies have demonstrated that MAP levels’ correlation to CO are volume state dependent, and thus CO might be a better parameter to focus on (4–6) since CBF depends on CO rather than on MAP in areas with impaired autoregulation (7). Further influencing factors may change CO levels, for example, atrial fibrillation or medication (8).

We therefore hypothesized that CP, assessed at bedside *via* perfusion ultrasound, depends on CO as a systemic parameter of organ perfusion rather than on MAP.

## Materials and Methods

### Subjects

Ten consecutive patients admitted to the Neurological Intensive Care Unit of University Hospital in Freiburg who were diagnosed with large ischemic stroke of the middle cerebral artery (MCA) territory *via* CT or MRI were included in this observational, prospective pilot study. Patients with other intracerebral lesions, known severe CO failure, and allergy to sonographic contrast were excluded. For clinical status and follow-up, patients’ symptom severity was assessed *via* the National Institute of Health Stroke Scale (NIHSS) and the modified Rankin Scale (mRS) on admission and at discharge. Additional to standard monitoring, all patients received an advanced hemodynamic monitoring with the parameters described below. Following hemodynamic baseline measurements, all patients received transcranial color-coded duplex sonography (TCCD) and transcranial perfusion sonography (TPS) according to a standardized protocol. Hemodynamic parameters then were optimized according to the target MAP and cardiac index (CI)-levels. Sonographic measurement was repeated after normalizing hemodynamic levels.

### Hemodynamic Assessment and Therapeutic Measures

As part of the in-house standard protocol for patients with large stroke, all patients received advanced cardiac monitoring either with a Vigileo- or a PiCCO-hemodynamic monitoring system (Flotrac-System, Edwards Lifesciences, USA; Pulsion Medical System, Germany) besides standard hemodynamic monitoring of heart frequency and MAP. These monitoring systems perform an analysis of the arterial pulse contour and calculate the CI (index results from CO and stroke volume related to body surface). Further monitoring parameters are the stroke volume variance (SVV; a dynamic parameter that reflects intravasal volume status) and the SVR-index [SVRI; a parameter identifying constriction or relaxation of systemic vasculature; (4)]. MAP levels of ≥70 mmHg and CI ranging between ≥3.0 and ≤4.5 were targeted. In patients with MAP < 70 mmHg or CI < 3.0, crystalloids should be administered as a first step until SVV < 10%. If this measure does not increase the hemodynamic parameters, noradrenaline, a peripheral vasoconstrictor, according to SVRI levels should be used. Dobutamine, as a positive inotropic substance, can be used as a last option in order to increase CI.

### Transcranial Sonography

#### Transcranial Color-Coded Duplex Sonography

Intracranial vascular status was assessed with a GE Logique 7 expert system (GE Healthcare, USA) using a 2 MHz-transducer focusing on the transtemporal approach in a meato-orbital plane. Mean MCA velocity (MCAv) was registered to determine the vessel recanalization status as well as perfusion status according to Doppler flow velocity.

#### Transcranial Perfusion Sonography

Transcranial perfusion sonography was performed bilaterally in the axial thalamic plane. For TPS, a harmonic mode with coded pulse inversion sequences was used (Pulsed sequences at 2 MHz and detection at 3 MHz, mechanical index 1.1–1.3, gain and transmit power was optimized individually in each patient, frame rate 1 Hz). Performance of TPS relied on the application ultrasound contrast media SonoVue (Bracco^®^), which consists of microbubbles containing sulfohexaflourane gas and coated with a phospholipid shell. To evaluate CP, we used a bolus-kinetic method with a high mechanical index, which aims to destroy the injected microbubbles that have entered microcirculation and lie in the field of the ultrasound beam. Time-triggered cineloops (45 s) of the cerebral image in the thalamic plane were obtained with an image frame rate of 1/1,000 ms. The resulting signals obtained over time represent time–intensity curves of ultrasound contrast media within the microcirculation and thus may detect regions of lacking or showing slowed CP. Obtained cineloops were then stored on an external work station for further offline analysis. For offline analysis, regions of interest (ROI) within the affected as well as the non-affected hemisphere were defined in the cortex, thalamus, and the basal ganglia. Time-to-peak (TTP) curves of the contrast media within the ROI were derived as surrogate marker of the CP and used for further analysis.

### Statistics

Obtained TTP data as well as MCAv were correlated with hemodynamic parameters (data at baseline and after optimizing hemodynamics). Continuous data was analyzed *via* Student’s *t*-test or Mann–Whitney *U* test according to the distribution level. Pearson’s correlation analyses were performed in order to reveal correlations of CI and MAP levels on one side and TTP and MCAv on the other side. Statistics were performed using IBM SPSS statistics 21 software.

## Results

Patients’ characteristics are outlined in Table 1. NIHSS levels did not change significantly from admission status (median 15.5 points) to time of discharge (median 16 points). mRS levels were stable (median 5.0 on admission and at discharge; three patients had died). Only four patients were admitted with an observed point of time of symptom onset (mean 153.5 ± 117.3 min before admission). The other six patients suffered from wake-up strokes. Hemodynamic monitoring was performed for 152.1 (± 128.9) h. It took 2.75 ± 5.9 h to optimize CI and until the second performance of TPS. When hemodynamics were optimized, MAP levels ranged from 73 to 110 mmHg and CI levels between 2.1 and 5.9. There was only one patient with atrial fibrillation as a possible influencing factor on CO but the patient showed constant optimal CI levels that did not make hemodynamic measures necessary. For optimizing CI levels, two patients received a volume challenge and additional inotropic therapy, two patients received singular volume challenge, and one patient received inotropic therapy alone. All other patients had no need for further therapeutic intervention concerning systemic hemodynamics.

**Table 1:** Patients’ characteristics.

Patient number	Age (years)	Gender	Vessel occlusion	Side	Treatment	Atrial fibrillation	Infarction etiology
1	64	Male	MCA + PCA	Right	None	No	Unknown
2	70	Male	ICA + MCA	Left	None	No	Atherosclerosis
3	60	Female	MCA + ACA	Bilateral	None	No	Other
4	45	Male	ICA + MCA + ACA	Right	IV-T	No	Other
5	46	Male	ICA + MCA + ACA	Right	None	No	Unknown
6	25	Female	MCA	Left	IV-T + EVT	No	Unknown
7	74	Male	MCA	Right	None	Yes	Cardioembolism
8	58	Male	ICA + MCA	Right	None	No	Unknown
9	54	Male	MCA	Left	None	No	Atherosclerosis
10	73	Female	None	Bilateral	None	No	Cardioembolism

*Vessel occlusion: internal carotid artery (ICA), middle cerebral artery (MCA), anterior cerebral artery (ACA), posterior cerebral artery (PCA); treatment: intravenous thrombolysis (IV-T), endovascular treatment (EVT); infarction etiology according to Trial of Org 10172 in Acute Stroke Treatment-criteria (9)*.

**Figure 1:**
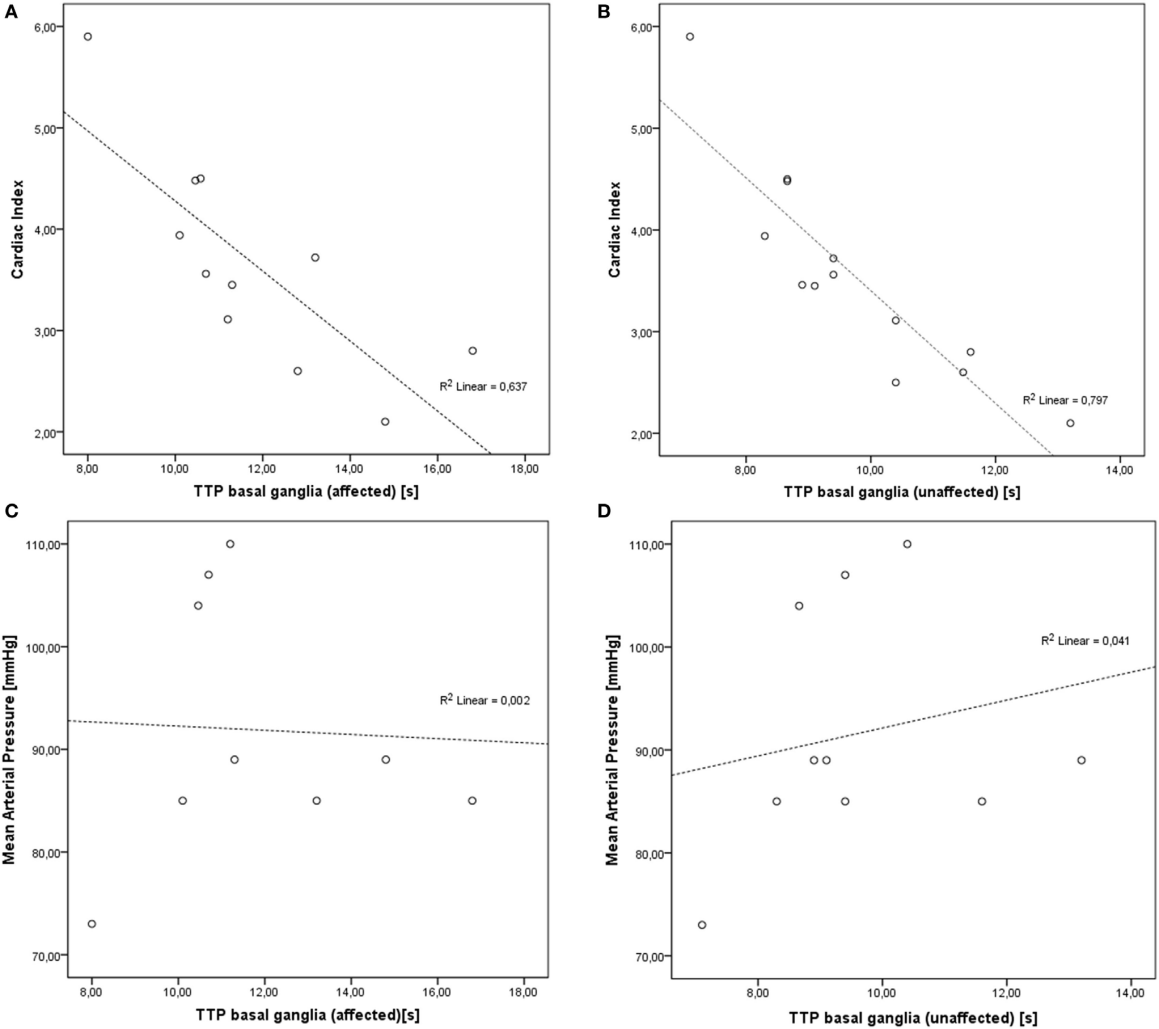
Correlations of cardiac index (CI) and mean arterial pressure (MAP) levels and time-to-peak (TTP) values. **(A)** Inverse correlation of CI and TTP in affected basal ganglia (*r* = −0.798, *p* < 0.001). **(B)** Inverse correlation of CI and TTP in unaffected basal ganglia (*r* = −0.893, *p* < 0.0001). **(C)** No significant correlation of MAP and affected basal ganglia (*r* = −0.044, *p* = 0.911). **(D)** No significant correlation of MAP and unaffected basal ganglia (*r* = 0.202, *p* = 0.575).

**Figure 2:**
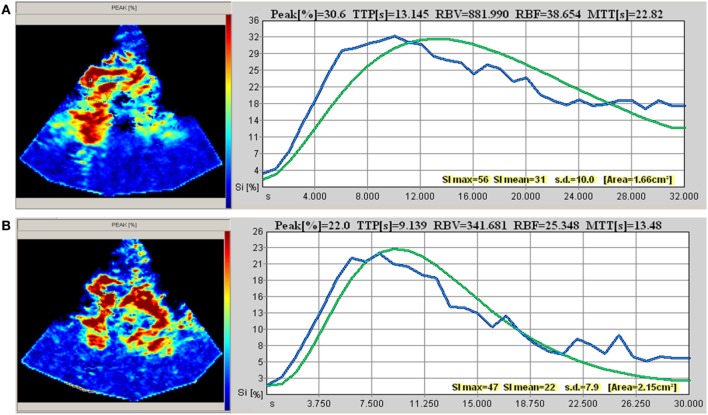
Transcranial perfusion sonography (example taken from patient 2). **(A)** Baseline measurement [before optimization of cardiac index (CI)] at CI 2.1 and mean arterial pressure (MAP) 89 mmHg. **(B)** Second measurement (after optimization of CI) at CI 3.45 and MAP 89 mmHg.

Correlation analyses of hemodynamic (at baseline and after optimization) and sonographic parameters showed various aspects: analyses of CI and MAP levels, TTP and MCAv revealed highly significant inverse correlations of CI and TTP in the affected and unaffected basal ganglia with *r* = −0.798 (*p* < 0.001) and *r* = −0.893 (*p* < 0.0001), respectively. In none of the patients, sufficient perfusion curves of the affected cortex could be obtained. No correlation was found for TTP with MAP (Figures 1 and 2).

No significant correlation of MCAv with CI (*r* = 0.552, *p* = 0.123) or MAP (*r* = −0.591, *p* = 0.094) was detected.

## Discussion

The results of this prospective pilot study challenge the current state of the art guiding hemodynamic therapy by MAP levels since they demonstrate that CP can rather be improved by CO-guided than MAP-focused hemodynamic therapy.

In healthy subjects, CBF is maintained constant independently of blood pressure changes by cerebral autoregulation (10). However, in acute stroke, autoregulation is injured in the ischemic and penumbral area, and furthermore, patients may suffer from global autoregulatory dysfunction (3, 11). The changes in MAP may lead to penumbral hypo- or hyperperfusion (10). Maintaining particularly penumbral perfusion in order to reduce the final infarction volume is thus the focus of therapeutic measures in stroke patients. Consequently, early penumbral reperfusion leads to improved patient outcomes (1).

Mean arterial pressure is the most widely used routine monitoring parameter in stroke (3, 12). Interestingly, in ischemic dysregulated areas, CP also depends on CO (7). Furthermore, physiological background claims organ oxygen delivery depending on CO (13). As the correlation of MAP and CO is volume state dependent, focusing on MAP in order to improve perfusion and oxygen delivery may hold true only in the isovolemic state (5, 6). Our preliminary data show that correlation of CP measured by the surrogate marker of TTP times examined by TPS with MAP is only moderate and not significant within the present sample size. Thus, optimizing MAP not necessarily implicates improvement of perfusion, and our data suggest CP being rather dependent on CO since we could demonstrate inverse correlation of TTP times with CO.

A preclinical trial with rats supports the implication as change of MAP by administration of phenylephrine showed no effect on infarction size or final functional outcome (14). Furthermore, other studies have shown a CO-dependent increase of CP in patients with subarachnoid hemorrhage (15). Various experimental interventions intending a shift of blood volume lead to a significant higher perfusion in rats with MCA-occlusion (14) and humans (5). Also, volume substitution lead to CO changes leaving MAP levels constant and secondary increased CP (15, 16).

The results of the present study should be regarded as preliminary due to its low patient number. We also did not apply quantitative radiographic methods for estimation of focal perfusion. Yet, TCCD has been found to be a reliable method to detect intracranial arterial pathologies before (17), and TPS is a sufficient technique to display infarction core and hypoperfused tissue at risk in ischemic strokes (18). Moreover, it could be performed at bedside in these critically ill patients.

We conclude that CO-dependent changes of CP may be more relevant than MAP-guided hemodynamic therapy in ischemic stroke patients in order to maintain and optimize cerebral penumbral perfusion. The clinical benefit of CO-guided therapy needs to be proven in further clinical trials.

## Ethics Statement

This study was carried out in accordance with the recommendations of the ethics committee of Freiburg University, Germany, with written informed consent from all subjects or their legal guardians. All subjects gave written informed consent in accordance with the Declaration of Helsinki. The protocol was approved by the ethics committee of Freiburg University, Germany.

## Author Contributions

HF contributed to the manuscript by doing the analysis and interpretation of data, drafting the article. MR and W-DN made substantial contribution to conception and design of the study, acquisition of data and revised the article. HF, MR, and W-DN made the final approval of the manuscript and agreed to be accountable for all aspects of the work.

## Conflict of Interest Statement

None of the authors received any financial compensation for their contribution to this study. W-DN and HF have received travel expense funding of Fresenius and AbbVie, respectively. MR has served on an advisory board for Daiichi Sankyo Inc. and has received speaker honoraria from Bayer Healthcare and Boehringer Ingelheim.
